# Blood cultures and blood microbiota analysis as surrogates for bronchoalveolar lavage fluid analysis in dogs with bacterial pneumonia

**DOI:** 10.1186/s12917-021-02841-w

**Published:** 2021-03-23

**Authors:** A. I. Vientós-Plotts, A. C. Ericsson, H. Rindt, C. R. Reinero

**Affiliations:** 1 Department of Veterinary Medicine and Surgery, College of Veterinary Medicine, University of Missouri, Columbia, MO 65211 USA; 2 Comparative Internal Medicine Laboratory, University of Missouri, Columbia, MO 65211 USA; 3 University of Missouri Metagenomics Center, University of Missouri, Columbia, MO 65211 USA; 4 Department of Veterinary Pathobiology, College of Veterinary Medicine, University of Missouri, Columbia, MO 65211 USA

## Abstract

**Background:**

Diagnosis of canine bacterial pneumonia relies on airway lavage to confirm septic, suppurative inflammation, and a positive bacterial culture. Considering risks of bronchoalveolar lavage fluid (BALF) collection, minimally invasive methods like culture or next generation sequencing of blood would be appealing. In dogs with bacterial pneumonia, our study aims included (1): determining proportion of agreement between cultivable bacteria in BALF and blood (2); characterizing BALF, blood, and oropharyngeal (OP) microbiota and determining if bacteria cultured from BALF were present in these communities; and (3) comparing relatedness of microbial community composition at all three sites. Bacterial cultures were performed on BALF and blood. After DNA extraction of BALF, blood and OP, 16S rRNA amplicon libraries were generated, sequenced, and compared to a bacterial gene sequence database.

**Results:**

Disregarding one false positive, blood cultures were positive in 2/9 dogs (5 total isolates), all 5 isolates were present in BALF cultures (16 total isolates). Based on sequencing data, all sites had rich and diverse microbial communities. Comparing cultured BALF bacterial genera with sequenced taxa, all dogs had ≥1 cultured isolate present in their microbiota: cultured BALF isolates were found in microbiota of BALF (12/16), blood (7/16), and OP (6/11; only 7 dogs had OP swabs). Of 394 distinct taxa detected in BALF, these were present in 75% OP and 45% blood samples. BALF community composition was significantly different than OP (*p* = 0.0059) and blood (*p* = 0.0009).

**Conclusions:**

Blood cultures are insensitive but specific for cultured BALF bacteria in canine bacterial pneumonia. Cultivable BALF bacteria were present in BALF, blood and OP microbiota to differing degrees.

## Background

Bacterial pneumonia, a common and serious respiratory disorder in dogs, is associated with substantial economic expense, morbidity, and in severe cases, mortality. The criterion standard for diagnosis and for optimal selection of antimicrobial treatment uses bronchoalveolar lavage fluid (BALF) to confirm septic, suppurative inflammation, and provide an antimicrobial susceptibility panel from a positive bacterial culture. One of the challenges presented to veterinary practitioners is that BALF collection requires general anesthesia, which may be particularly risky in patients with respiratory compromise. Less invasive sampling procedures such as oropharyngeal (OP) swabs or trans-tracheal wash, either do not reliably correlate with BALF results or are feasible only in large dogs, respectively [1–3].

Inability to obtain a sample for culture usually results in use of empiric broad-spectrum antimicrobials. Since the standard of practice in veterinary medicine is to treat dogs with bacterial pneumonia for 3–6 weeks or more [4], if the organism is not susceptible to the empiric antimicrobial selected, this may lead to disease progression or development of antimicrobial resistance. The development of a minimally invasive method (e.g., blood draw) to identify the causative agent of bacterial pneumonia could provide a means to overcome this challenge. In human medicine, blood cultures may help identify the causative agent of bacterial pneumonia in patients with moderate to severe clinical signs [5, 6]. The first aim of this study was to determine the proportion of agreement between cultivable bacteria in BALF and blood in dogs with bacterial pneumonia. Assuming agreement between these diagnostic tests, minimally invasive blood cultures could serve as surrogates for BALF culture.

While there is admittedly potential with optimized collection protocols for detection of microbes in blood cultures, they are currently overall considered a low yield diagnostic test in people [7, 8]. However, advances using culture-independent methods of microbial identification, such as PCR and more recently, bacterial 16S rRNA gene sequencing, have allowed for higher sensitivity and specificity in the detection of organisms [9]. Prior to the development of these techniques, sites like the lungs and blood had previously been considered sterile. Studies in humans [10, 11], and a variety of animals including dogs [12], cats [13], sheep [14], cattle [15], horses [16] and mice [17] demonstrate the presence of resident bacterial populations in the respiratory tract, and that alterations in these populations (i.e., dysbiosis) are associated with disease. The second aim of this study was to characterize and compare the relatedness of BALF, blood and OP microbial community compositions, and then determine if there is a correlation between the predominating taxa found in sequencing analysis with the bacterial isolates identified via BALF culture**.** Characterization of bacterial populations using 16S rRNA sequencing may have clinical applications, such as more specific identification of bacteria and identification of non-cultivable organisms. While once considered too cumbersome for practical clinical application, emerging platforms show promise to provide rapid, bedside tests for specific pathogens [18] and the lung microbiota in general [19].

To determine the utility of blood cultures and sequencing of blood-derived DNA as tools in the diagnosis of bacterial pneumonia, nine dogs were prospectively enrolled in this study. We hypothesized that companion dogs with bacterial pneumonia would have low numbers of positive blood cultures but that bacteria cultured from blood would correlate with bacteria cultured from the lungs. As next generation sequencing is a powerful tool to characterize the microbiota of BALF, blood and OP and allow for identification of cultivable bacteria within these microbiota, we further hypothesized there would be a correlation between predominating taxa in the microbiota to the causative agent of bacterial pneumonia identified via BALF culture.

## Results

Twenty-four dogs were screened, nine were enrolled, and 15 were excluded due to recent history of antibiotic administration (*n* = 8), recent surgery (2), and a body weight < 8 kg (5). Enrolled dogs included 6 female (1 intact, 5 spayed), and 3 male neutered dogs, with a mean age of 4.6 years ranging from 4 months to 13 years old and weighing a mean of 33 kg (20–65 kg). Dogs from a variety of breeds were enrolled including Irish wolfhound (*n* = 2), German shepherd (2), Labrador retriever (2), golden retriever (1), standard poodle (1) and mixed breed (1). All dogs had at least one clinical sign including cough (*n* = 7) fever (6), tachypnea (3), regurgitation (3), ptyalism (1), hematemesis (1), and hemoptysis (1). CBCs showed peripheral neutrophilia (*n* = 5) or neutropenia (1); bands or neutrophil toxicity were common (7). Cytology of BALF demonstrated degenerate neutrophils (*n* = 7) and intracellular bacteria (4) with all dogs having at least one of those findings. All nine dogs had a positive BALF culture.

Dogs were classified as having community-acquired pneumonia (CAP, dogs 1 and 2) or aspiration pneumonia (AP, dogs 3 to 9). Predisposing factors or underlying defects in dogs with AP included megaesophagus (*n* = 2), vomiting (2), dysphagia due to a salivary gland mucocele (1), near drowning in sewage water (1), and geriatric onset laryngeal paralysis and polyneuropathy (1). A single organism was identified from BALF cultures of both dogs with CAP and 3/7 dogs with AP. The remainder of the dogs with AP had more than one organism identified on BALF culture. Three dogs (2, 6 and 7) were euthanized; the remaining six dogs recovered completely.

Bacteria isolated from BALF and blood cultures are listed in Table 1 (columns 2–3). While all BALF cultures isolated at least one bacterial species, blood cultures failed to yield any organisms in 6 dogs and grew a likely skin contaminant in 1 dog (*Staphylococcus pseudintermedius,* dog 8). Of the 2 dogs with true positive blood cultures, those same bacterial species were found in BALF cultures. One of the dogs with bacteremia (dog 7), was the only one in the study with a clinical picture consistent with sepsis, which included neutropenia, fever, and lethargy. Dogs 8 and 9 had a cough, anorexia and mild neutrophilia.

**Table 1:** Match of bacterial taxa between BALF and blood (culture results versus most abundant bacteria from microbiome analysis)

Dog#	BALF Culture Result		Blood Culture Result	Most abundant genus (% RA)					
	Genus	Species		BALF		Blood		OP	
1^a^	*Haemophilus*^*b*^	*haemoglobinophilus*	No growth	*Haemophilus*^b^	(23.4)	*Mycoplasma*	(37.8)	*Haemophilus*	(26.2)
2^a^	*Pasteurella*	*canis*	No growth	*Acinetobacter*	(19.4)	*Streptococcus* sp.	(18.5)	*Frederiksenia*^*c*^	(31.7)
3	*Pseudomonas*	*putida*	No growth	*Brevundimonas*	(32.7)	*Gemmata*	(12.9)	*Haemophilus*	(68.0)
	*Stenotrophomonas*	*maltophila*							
	*Achromobacter*	*xylosoxidans*							
4	*Pseudomonas*	*putida*	No growth	*Carnobacterium*	(69.9)	*Streptococcus*	(23.6)	N/A	
	*Pseudomonas*	*oleovorans*							
5	*Lactobacillus*	*salivarius*	No growth	*Lactobacillus*	(56.2)	*Cutibacterium*	(30.7)	*Lactobacillus*	(21.7)
6	*Escherichia*^b^	*coli*	No growth	*Escherichia*^b^	(97.5)	*Streptococcus*	(13.4)	*Haemophilus*	(17.1)
7	*Escherichia*^b^	*coli*	*E. coli*^b^	*Escherichia*^b^	(91.6)	*Streptococcus*^b^	(15.4)	N/A	
	*Klebsiella*^b^	*pneumoniae*	*Klebsiella pneumoniae*^b^						
	*Streptococcus*^b^	*canis*	*Streptococcus canis*^b^						
8	*Klebsiella*	*oxytoca*	*Staphylococcus pseudintermedius*	*Acinetobacter*^b^	(55.8)	*Acinetobacter*^b^	(83.9)	*Alloprevotella*	(20.7)
	*Streptococcus*	*canis*							
9	*Neisseria*^b^	*weaveri*	*Neisseria weaveri*^c^	*Frederiksenia*^*c*^	(37.1)	*Acinetobacter*	(61.6)	*Frederiksenia*^*c*^	(24.3)
	*Ursidibacter*^d^	*maritimus*	*Federiksenia canicola*						

*BALF* bronchoalveolar lavage fluid, *OTU* operational taxonomic unit, *RA* relative abundance, *N/A* not available (OP not collected)

^a^ Community-acquired pneumonia; ^b^ Represent identification of the same taxa via culture and 16S rRNA sequencing; ^c^ Frederiksenia sp. at the genus level comprised by a single OTU: *Pasteurellaceae* bacterium Orientalotternb1; ^d^ A *Pasteurella* like organism with no known pathogenic potential

**Fig. 1:**
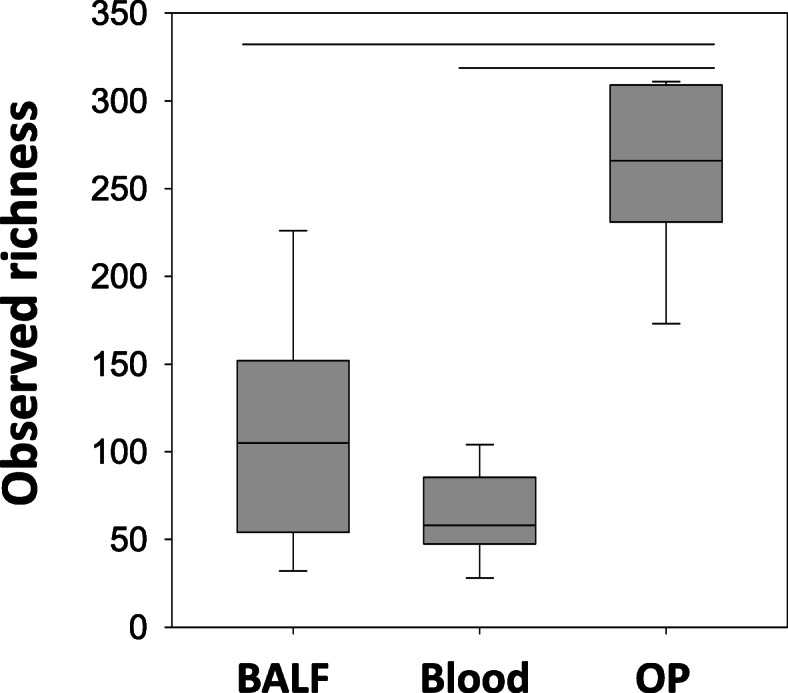
Box and whisker plot displaying richness (total number of unique taxonomic units in a sample) observed at each site. The black bars in the box structures represent the median value, the bottom line of the box to the black line represents the first quartile and the black line to the outer box line represents the third quartile. The line extended vertically from the box (both on the bottom and the top) to the solid horizontal line, represents the extent of the area where outliers are found. Richness was significantly higher in oropharyngeal swabs (OP) compared to bronchoalveolar lavage fluid (BALF) (*p* = 0.018) and blood (*p* < 0.001)

**Fig. 2:**
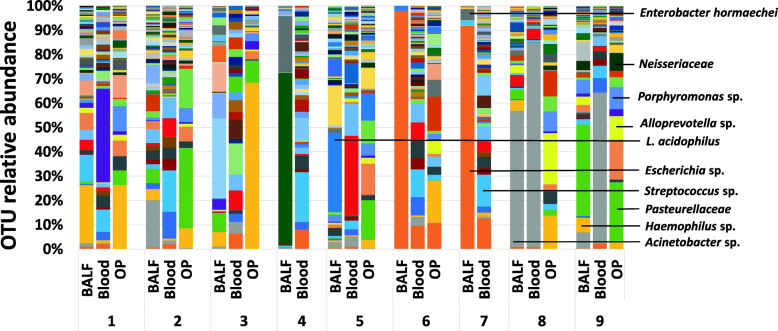
Bar graph displaying the percentage relative abundance of different bacterial taxa at the level of the operational taxonomic unit (OTU) in bronchoalveolar lavage fluid, oropharyngeal swabs and blood. Each color represents a different OTU and with all OTUs from a single sample (BALF or blood) adding up to 100%. Numbers at the bottom of the graph represent a different dog

In parallel with bacterial cultures, the bacterial microbiota was characterized using culture-independent 16S rRNA amplicon sequencing. Following amplification and sequencing, the total number of high-quality sequences detected (i.e., coverage) varied by site. Compared with BALF, blood had significantly lower coverage [median, (range)] 2711 (276–284,412) and 375 (62–1449) sequences, respectively; *p* = 0.006]. Coverage was also significantly higher in OP samples [216,580 (88,649 – 273,962)] compared to blood (*p* < 0.0001), but not significantly different compared to BALF (*p* = 0.211). The richness, defined as total number of unique operational taxonomic units (OTUs, groups of sequences sharing a minimum of 97% nucleotide identity) of BALF was also higher, but not significantly, than that of blood: the median number of OTUs was 105 (32–226) in BALF and 58 (28–104) in blood; *p* = 0.112. The richness in OP samples [266 (173–311)] was significantly higher than BALF (*p* = 0.018) and blood (*p* < 0.001) (Fig. 1). The relative abundance (RA) of all taxa detected in BALF, blood and OP is shown in Fig. 2.

Comparisons were made between cultured BALF bacteria with sequenced taxa present to any degree in the microbiota of the BALF, blood and OP (Table 2). Twelve out of 16 cultured isolates were found in the microbiota of BALF at the level of the genera ranging from 0.05 to 97.5% relative abundance. All dogs (9/9) had at least one cultured BALF isolate reflected in the BALF microbiota, and 5/9 dogs had all of the BALF cultured bacteria detected in BALF sequencing. Seven out of 16 BALF isolates were found in blood microbiota ranging from 0.2 to 15.5% relative abundance. In the seven dogs with OP swabs, 6/11 BALF isolates were found in the OP microbiota ranging from 0.3 to 26.2% relative abundance, and 6/7 dogs had at least one of the BALF cultured bacteria identified in OP sequencing. Comparisons were also made between the most abundant bacterial taxa at the level of the genera identified in BALF, blood and OP sequenced samples (Table 1 (columns 4–6)). When comparing only BALF cultures and BALF sequencing results, there was agreement between taxa identified via culture and the most abundant OTU in 4/9 dogs (44%). Comparisons of bacterial species identified from blood cultures with BALF cultures or with BALF and blood microbiotas were challenging due to the low number of positive blood cultures. In the 2 dogs with positive blood cultures, the cultured bacteria were different from the genera found at the highest RA in the blood microbiota; however, of the 5 bacterial isolates cultured from blood in these 2 dogs, 3 were found at a lower relative abundance in the blood microbiota. Only 1 of 9 dogs (dog 7) had agreement between bacteria identified in the BALF culture, blood culture and the most abundant OTU in BALF sequencing results. There was agreement between the predominant OTU identified in OP swabs and the organism identified on BALF culture in dogs 1 and 2 having CAP. In these two dogs, the most abundant OTU in OP was also present in the BALF microbiota. In five dogs with AP in which OP swabs were obtained, the most abundant OTU in the OP was not cultured from BALF nor was it present in the BALF microbiota.

**Table 2:** Percent relative abundance of taxa identified via culture present in lower airways, blood and oropharyngeal swab sequencing at the genus level

Dog #	BALF Cultured Genus	%RA of cultured BALF genus in sequencing		
		BALF	Blood	OP
**1**	*Haemophilus*	23.4	1.2	26.2
**2**	*Pasteurella*	6.7	0.20	1.2
**3**	*Pseudomonas*	11.9	0	0
	*Stenotrophomonas*	3.8	0	0
	*Achromobacter*	0	0	0
**4**	*Pseudomonas putida*	1.9	0	N/A
	*Pseudomonas oleovorans*			
**5**	*Lactobacillus*	56.2	0.77	21.8
**6**	*Escherichia*	97.5	9.5	10.7
**7**	*Escherichia*	91.7	12.5	N/A
	*Klebsiella*	0	0	N/A
	*Streptococcus*	2.2	15.5	N/A
**8**	*Klebsiella*	0	0	0
	*Streptococcus*	2.4	1.2	6.3
**9**	*Neisseria*	0.05	0	0.3
	*Ursidibacter*	0	0	0

*BALF* bronchoalveolar lavage fluid, *OP* oropharynx, *RA* relative abundance, *N/A* not available (OP not collected)

The lower airways of two of the four dogs that showed agreement between BALF culture and sequencing were dominated by one organism with the most abundant OTU comprising > 90% of the bacterial community (Fig. 2, dogs 6 and 7). Predominance of one organism in the microbiota was also noted in dogs 4 and 8 despite having no agreement between BALF culture and sequencing (Fig. 2). Collectively, these 4 dogs showed marked lower airway dysbiosis, as evidenced by the overall decrease in diversity. Interestingly, the 2 dogs that had growth on blood culture also showed dominance of one OTU annotated as *Acinetobacter* composing 84 and 62% of the blood microbiota in dogs 8 and 9 respectively. In OP, dominance of one OTU was observed in dog 3, with *Haemophilus spp.* composing 68% of the upper airway microbiota.

**Fig. 3:**
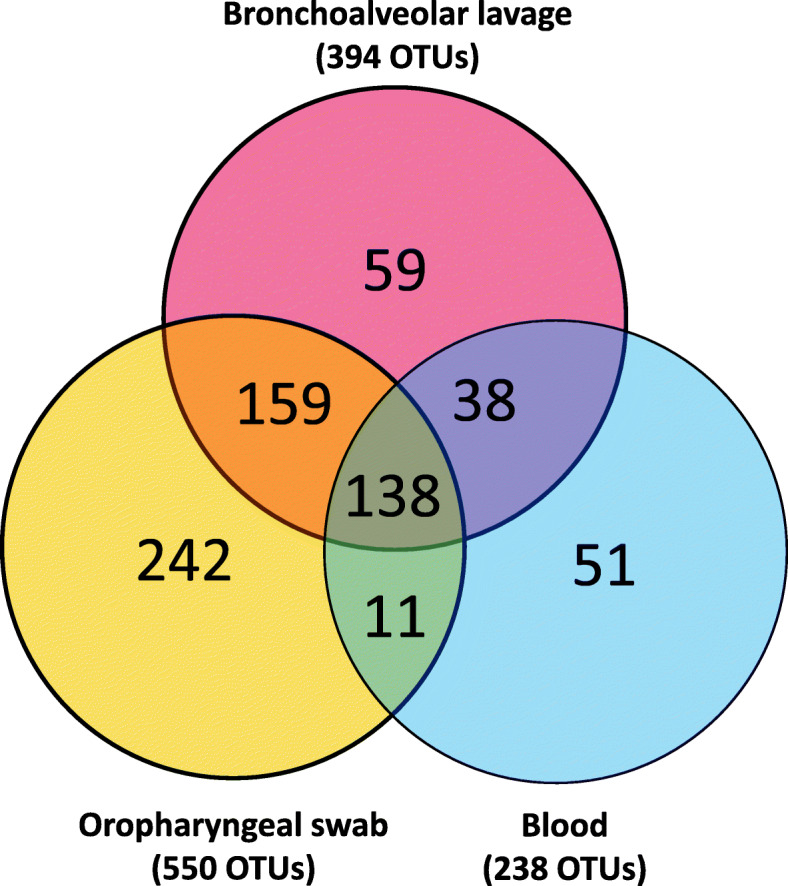
Venn diagram showing the distribution of operational taxonomic units (OTUs) detected in at least one sample of bronchoalveolar lavage fluid (BALF), oropharyngeal swab (OP) or blood of dogs with bacterial pneumonia

Patterns of co-occurrence of OTUs (i.e., presence or absence of distinct taxa) between different sample sites as shown in a Venn diagram support some degree of regional continuity (Fig. 3). Of the 394 distinct OTUs found in BALF, 297 (75%) were detected in OP and 176 (45%) were detected in blood. Only 51 of 238 (21%) distinct OTUs in blood failed to be represented in either the upper or lower airway samples.

**Fig. 4:**
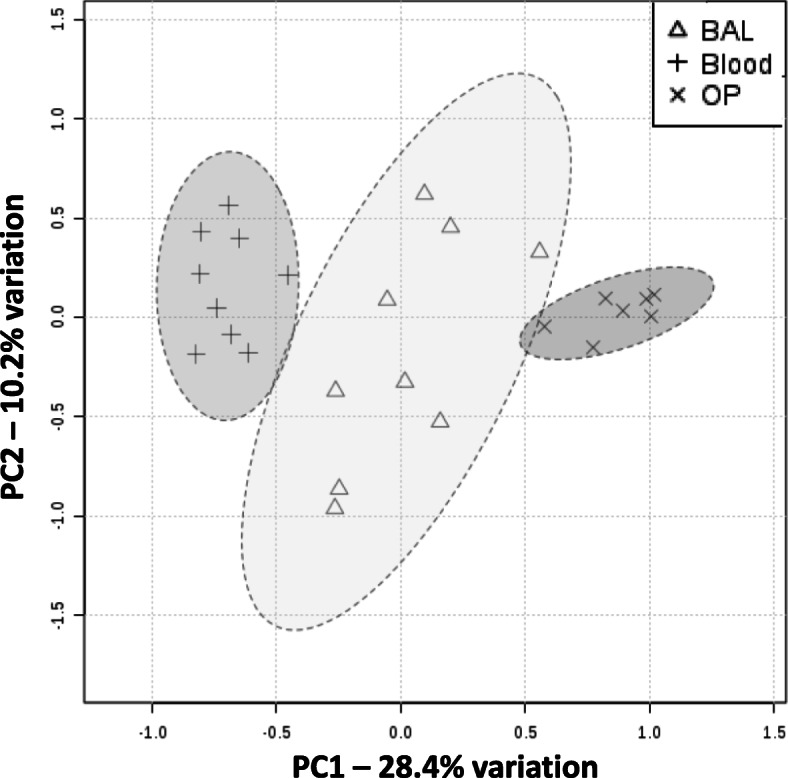
β-diversity as shown via principal component analysis. Unweighted principal component analysis of samples from all sample sites (BALF, OP and blood) PC1 versus PC2; legends at right. The ellipses represent 95% intervals

Principal Component Analysis (PCA) was used to examine community sample relatedness (i.e., β-diversity). As shown in Fig. 4, there is minimal overlap between BALF and OP, and neither site overlapped with blood with a 95% confidence interval. PERMANOVA of the Bray-Curtis similarity index found that the bacterial community composition in BALF was significantly different than OP (*p* = 0.0059) and blood (*p* = 0.0009), and OP was significantly different to blood (*p* = 0.0001).

## Discussion

Reliance on culture of BALF, but not blood, to identify clinically relevant bacterial pathogens in dogs with bacterial pneumonia was supported in this study. While less invasive, blood cultures were infrequently positive, limiting their utility as a surrogate for BALF cultures. Cultured BALF bacterial isolates were more frequently identified in BALF sequencing compared to blood or OP. Additionally, although there was overlap, the BALF microbial community composition was significantly different than blood and OP.

The recent antimicrobial use guidelines on respiratory tract infections generated by the International Society of Companion Animal Infectious Disease (ISCAID) [20] reached a consensus that in dogs with severe pneumonia, blood cultures should be considered as an alternative to airway lavage in high-risk patients despite the lack of published studies comparing BALF and blood isolates in dogs with bacterial pneumonia. Based on the current study in which the majority of blood cultures were negative, blood cultures appear to be an insensitive diagnostic for detection of clinically relevant bacteria in dogs with bacterial pneumonia. This is consistent with studies in children with any pneumonia-associated complication including sepsis and organ dysfunction, showing positive blood culture rates ranging from 2 to 19% [21, 22]. The low positivity rate observed in blood cultures, in dogs and humans, may be a function of the low biomass of blood. One of three blood cultures was strongly suspicious for a skin contaminant (*Staphylococcus pseudintermedius*), underscoring the need to interpret culture results with the clinical picture. However, 2/3 positive blood cultures were in agreement with BALF culture suggesting positive blood cultures appear to be specific for the bacterial pathogen causing pneumonia. This finding will need to be repeated in larger studies.

In comparison to routine use of bacterial cultures to guide therapeutic management of bacterial pneumonia, 16S rRNA gene sequencing is not currently used clinically. Little is known about the role of complex, diverse microbial communities in the lung and how disease results in perturbation of these communities. Until recently, the healthy lung was assumed to be sterile and cultivable bacteria in patients with bacterial pneumonia were presumed to be an invading pathogen causing disease [23–25]. With recent documentation of a rich and diverse healthy canine microbiota [12], it is now understood there is a more complex bacterial microenvironment in the respiratory tract. The respiratory microbiota may influence and be influenced by the microbiota at other sites including the blood and upper airways. The regional continuity observed between the upper and lower airways, as evidenced by the 75% overlap between the OTUs identified in the BALF compared to OP, suggest there is a robust relationship between the microbiota at distinct sites of the respiratory tract.

This study underscores the importance of understanding how cultivable bacterial species assumed causative of pneumonia interplay with the microbiota. Not all cultured bacteria are causative of disease and interpretation of culture results must be performed in light of the entire clinicopathologic picture and knowledge of putative pathogens. In the current study, the blood culture revealing *Staphylococcus pseudintermedius*, a known skin contaminant [26], is likely unrelated to the respiratory infection. Although this organism may be found in primary infectious sites, it is not a reported cause of pneumonia. Bacteria which could be causative of disease may not be able to be cultured (or may be challenging to culture) with standard laboratory techniques. *Mycoplasma spp*., believed a common cause of canine infectious respiratory disease complex, are a good example of this [27]. Additionally, culture of a bacterial pathogen from BALF was not always associated with a dominating taxon identified in the respiratory microbiota. In this study, several patterns of the complex relationship between cultivable and sequenced bacteria were noted. One pattern revealed a predominant OTU that obliterated nearly all other bacterial communities. For example, sequencing of BALF in dogs 6 and 7 showed a predominance of *Escherichia* sp. with a relative abundance greater than 91.5%. This was not only reflected in the lower airways, but also in blood with two dogs having a positive blood culture and a predominance of the corresponding organism identified on blood sequencing. This pronounced loss of microbial diversity has been documented in humans with pneumonia on mechanical ventilators [28] and with cystic fibrosis [29]. It has also been described in a prior study of dogs with bacterial pneumonia [27]. A second pattern showed a correspondence of the cultured BALF bacteria with the most abundant OTU obtained via sequencing, without that OTU nearly eradicating the remainder of the microbiota. For example, this was seen in dogs 1 and 5 in which *Haemophilus sp.* comprised 23.4% and *Lactobacillus sp.* 32.6% of the sequenced bacteria, respectively. A third pattern was one in which the cultured bacteria from BALF was not reflected as the most abundant OTU, but was still present within the sequenced microbiota (Table 2). Future studies would be required to elucidate if, in this scenario, the cultured bacteria are not the true pathogens causative of pneumonia, and the microbiota reflects an uncultivable pathogen present at highest abundance. A final pattern was one in which the cultivable bacterial species was not present in the microbiota at all (Table 2).

Overall, cultivable bacterial species from BALF were reflected to some degree in the microbiota of BALF (9/9 dogs) and sometimes blood (5/9 dogs; even when blood cultures were negative). When assessing presence of cultivable bacteria in BALF (*n* = 16 species total), only 7 taxa were found at any abundance in the blood microbiota. Thus, using 16S rRNA gene sequencing of blood as a non-invasive sample does not appear widely useful as a surrogate for BALF. Similarly, in the 7 dogs in which OP samples were sequenced, only 6 taxa corresponding to 11 organisms cultured from BALF were found in any abundance in the OP microbiota. Additionally, it is important to note that in contrast with standard culture techniques, evaluation of the blood microbiome profile does not provide information in regard to the antimicrobial susceptibility. Taking this fact into consideration, evaluation of the microbiome may not be as clinically useful as a culture. However, it may provide data for research evaluating whether or not there exist a correlation between a specific microbiome profile and antimicrobial response, or identification of antimicrobial resistance genes.

Interestingly, of the 3 dogs in this study had agreement between BALF cultures and sequencing at all 3 sites, 2 of those dogs had CAP. These two dogs grew *Haemophilus spp.* and *Pasteurella spp.*, respectively, from BALF cultures. Both of these organisms belong to the *Pasteurellaceae* family, which has been identified in BALF sequencing of healthy dogs [12]. Although neither of these organisms are considered contagious bacterial pathogens, many primary viral pathogens within CIRDC that are permissive for secondary bacterial infection. Viral infections are thought to cause ciliary impairment and facilitate bacterial adherence via upregulation of glycoproteins F and G, therefore contributing to a disruption of the homeostatic balance of resident microbial populations [30, 31].

It could be speculated that the blood microbiota is comprised of microbial populations from all organs including the gastrointestinal tract and respiratory tract, among others, and disease primarily affecting one organ system could result in altered blood microbiota. In general, the cultured BALF bacteria were present at low relative abundance (< 1.5%) in blood sequencing with the exception of the two dogs with marked predominance of one OTU in BALF (dogs 6 and 7) where relative abundance of cultured BALF bacteria were between 9.5 and 15.5%. These two dogs could represent instances in which alteration of the microbiota in the blood occurred secondary to respiratory dysbiosis. Similarly, although the upper and lower airway microbial communities are distinct, there appears to be evidence of regional continuity. Aspiration leads to translocation of upper airway bacteria into the lower airways, and lower airway bacteria can be translocated into the upper airway while coughing. This regional continuity has been previously described in healthy dogs [12], and appears to be more dramatic in dogs with pneumonia, which merits further investigation of OP microbial communities as surrogates for lower airway microbial communities.

## Conclusions

Identification of clinically relevant bacteria in dogs with CAP and AP will continue to rely on BALF and not blood cultures. However, if blood culture is performed and in the uncommon scenario a pathogen is identified, it is likely to reflect the causative species of bacterial pneumonia. Understanding the limitations of standard culture techniques, analysis of microbial communities using 16S rRNA sequencing of BALF, blood and OP identified many more taxa that could contribute to the development of disease, whether that is as a single pathogen overgrowth or other alterations in the microbial community composition.

## Materials and methods

### Animals

Client-owned dogs (*n* = 9) presenting to the University of Missouri Veterinary Health Center between August 2017 and September 2018 were prospectively enrolled in this descriptive observational case series if they were diagnosed with bacterial pneumonia not caused by systemic infection and fit the following inclusion and exclusion criteria. This study was performed in compliance with the University of Missouri Institutional Animal Care and Use Committee and Informed client consent was obtained prior to enrollment, in accordance with the protocol (IACUC # 8240). Dogs had to have a positive BALF bacterial culture with BALF cytology showing septic, suppurative inflammation with or without extra/intracellular bacteria. Other inclusion criteria were a baseline complete blood count, with or without evidence of systemic inflammation or infection and three-view thoracic radiographs with the presence of an interstitial, bronchial, alveolar or a combination of these. Dogs were excluded if they had (1) more than one dose of antibiotics or a single dose of long-acting cefovecin within 14 days of presentation (2), evidence of another identifiable infection likely to cause bacteremia such as an infected open wound, or history of recent surgery, or (3) a body weight < 8 kg. Blood culture media with resins (BD Bactec™ Plus aerobic/F culture vials, Becton, Sparks, MD) was used for culture in dogs having had a prior dose of antibiotic.

Dogs were subsequently subclassified as having community-acquired or aspiration pneumonia. Community-acquired pneumonia (CAP) was defined as infection occurring in an otherwise healthy dog with no underlying structural or immunologic defects; this term refers to pathogens associated with canine infectious respiratory disease complex (CIRDC) [32]. In addition to primary CIRDC bacterial pathogens (e.g., *Bordetella bronchiseptica*), primary CIRDC viral infections (e.g., canine distemper virus) leading to secondary bacterial infection by resident microbial populations were included in this definition. Aspiration pneumonia (AP) was defined as pneumonia occurring as a result of a predisposing factor or an underlying anatomic, physiologic or immunologic defect [4, 33]. As aspiration pneumonia can occur without bacterial infection, definitive confirmation of secondary bacterial pneumonia was required as described previously. All medical records were reviewed, and the following information was extracted: signalment, presenting clinical signs, results of a CBC and BALF cytology and culture, underlying cause of the bacterial pneumonia and outcome (recovery, death or euthanasia).

### Sample collection

Anesthetic protocols were performed at the discretion of a board-certified anesthesiologist. Using a sterilized endoscope, BALF was collected by instilling a 20 mL aliquot of sterile, warmed saline through the biopsy channel. A minimum of 2 mL was submitted for cytologic analysis, a minimum of 1 mL was submitted for bacterial culture, and ≥ 5 mL was banked for microbiota analysis. An aliquot of sterile saline was run through the scope as a negative control for culture and microbiota analysis prior to starting the endoscopic procedure. A subset of dogs (*n* = 7) also had OP swabs collected for microbiota analysis. Cotton swabs (Puritan, Hardwood Products Company, Guilford, Maine, 04443 USA) were autoclaved for 30 min in a glass container, and later used for OP sample collection. Briefly, a sterile swab was inserted into the oropharynx prior to intubation and placed in a sterile tube containing lysis buffer before DNA isolation. Three blood samples for culture were obtained from different vascular sites at least 30 min apart [34]. The collection sites were clipped and aseptically prepared prior to venipuncture. The maximum blood volume collected with each venipuncture was based on the weight of the patient: 8–12 kg (10 mL), 12–18 kg (12 mL), > 18 kg (14 mL). Blood volume was calculated to remain well under 8% of circulating blood volume, which is the maximum amount recommended to be removed without replacing the volume with crystalloid fluids [35].

Blood samples were inoculated into a soybean-casein digest broth containing resins to enhance recovery of organisms (BD BACTEC™ Plus Aerobic/F Culture Vials, Becton, Dickinson and Company, Sparks MD 21152 USA).

Four milliliters of blood were added to an EDTA tube to be banked for microbiota analysis, with the remainder of the blood equally divided between the aerobic and anaerobic culture bottles. The vials were maintained at room temperature for up to 12 h until arrival at the laboratory. Additional controls for microbiota analysis included samples obtained from a needle puncturing skin but not the blood vessel after aseptic preparation, and DNA extraction reagents. Promptly after collection, red blood cells were lysed, DNA was extracted as previously described [27] and samples were stored at − 20 °C until sequencing.

### BALF and blood cultures

Samples were submitted the University of Missouri Veterinary Medical Diagnostic Laboratory for aerobic and capnophilic testing. Ten μL calibrated loops were used to deliver aliquots of BALF to blood agar & MacConkey agar plates for aerobic cultures, and chocolate agar plates for anaerobic cultures. All aerobic samples were incubated at 35 °C, and capnophilic cultures were incubated at 35 °C with 95% air and 5% CO_2_ for 24 to 36 h. Blood culture bottles were incubated at 35 °C and observed daily for turbidity. If no turbidity developed, blind aerobic and anaerobic subcultures were performed. Aerobic culturing was accomplished using 5% Trypticase Soy Blood agar plates and MacConkey agar. Anaerobic cultures were performed with pre-reduced 5% Trypticase Soy Blood agar plates. Bacterial isolates were Gram stained and either identified with conventional biochemical reactions [36], the Automated Sensititre AP-80 or AP-90 for aerobic bacteria or MALDI-TOF identification system (Matrix Assisted Laser Desorption/Ionization-Time of Flight: Bruker Daltonics, Inc. 40 Manning Road, Manning Park, Billerica, MA 01821).

### DNA extraction

DNA was extracted using a manual nucleic acid precipitation, then resuspended in a buffer and purified using Qiagen DNeasy blood and tissue kits (850 Lincoln Centre Drive, Foster City, California, 94,404, USA) according to manufacturer’s instructions with minor modifications, as previously described [13]. Yields were determined via fluorometry (Qubit 2.0, Invitrogen, Carlsbad, CA) using Qubit dsDNA BR assays (Invitrogen). Samples were stored at − 20 °C until library preparation was performed.

### 16S rRNA library preparation and sequencing

Library construction and sequencing were performed at the University of Missouri DNA Core facility, as previously described [12]. Briefly, 16S rRNA amplicons were generated via amplification of the V4 region of the 16S rRNA gene using dual-indexed universal primers (U515F/806R) [37, 38] flanked by Illumina standard adapter sequences. Following amplification, products were pooled for sequencing using the Illumina MiSeq platform and V2 chemistry with 2 × 250 bp paired-end reads. Qiime v1.9.1 software [39] was used to perform de novo and reference-based chimera detection and removal, and remaining contiguous sequences were assigned to operational taxonomic units (OTUs) via de novo OTU clustering and a criterion of 97% nucleotide identity. Taxonomy was determined for selected OTUs using BLAST against the SILVA database [40, 41]. Principal component analyses (PCA) were performed using ¼ root-transformed OTU relative abundance data in PAST 3.18 [42]. Metrics of richness and α-diversity were determined based on a rarefied dataset subsampled to a uniform read count of 2289 reads per sample using beta_diversity_through_plots.py, available at http://qiime.org/scripts/beta_diversity_through_plots.html.

### Statistical analysis

Distribution of read counts in experimental and control samples was first tested for normality using the Shapiro-Wilk method, and differences in read count were then determined using an ANOVA on ranks, and post hoc analysis using Dunn’s Method due to non-normality, implemented in SigmaPlot 13.0. Differences in β-diversity between BALF, blood and OP swab communities were determined and compared using one-way permutational multivariate analysis of variance (PERMANOVA) of Bray Curtis similarity index implemented in Past 3.18 [42]. In all cases, significance was established as *p* < 0.05.

## Data Availability

All sequence data have been deposited in the NCBIA Sequence Read Archive (SRA) under the BioProject accession number: PRJNA628912 (https://www.ncbi.nlm.nih.gov/bioproject/?term=PRJNA628912).

## Abbreviations

BALF: Bronchoalveolar lavage
OP: Oropharyngeal swab
OTU: Operational taxonomic unit
RA: Relative abundance
